# Celluloid Base

**Published:** 1872-02

**Authors:** 


### CELLULOID BASE.

This material, introduced to the profession in the last two years, as now improved and manipulated, is a very valuable material for cheap dentures. It is easily worked, is as permanent in the mouth as rubber, and far less objectionable in many respects. It is being used by a great many in the profession, and is being introduced very rapidly. It affords an opportunity to every one to abandon the use of rubber, and have a better material, that will be more satisfactory to those who use it; and we suggest to every one in the profession to immediately take it up, and let go that other thing.

